# Publisher Correction: Influence of nutrient supply on plankton microbiome biodiversity and distribution in a coastal upwelling region

**DOI:** 10.1038/s41467-022-30665-1

**Published:** 2022-05-18

**Authors:** Chase C. James, Andrew D. Barton, Lisa Zeigler Allen, Robert H. Lampe, Ariel Rabines, Anne Schulberg, Hong Zheng, Ralf Goericke, Kelly D. Goodwin, Andrew E. Allen

**Affiliations:** 1 Scripps Institution of Oceanography, University of California San Diego, 9500 Gilman Dr, La Jolla, CA 92093 USA; 2 J. Craig Venter Institute, 4120 Capricorn Lane, La Jolla, CA 92037 USA; 3 Section of Ecology, Behavior and Evolution, University of California San Diego, 9500 Gilman Dr, La Jolla, CA 92093 USA; 4 Atlantic Oceanographic and Meteorological Laboratory, (Stationed at Southwest Fisheries Science Center), 4301 Rickenbacker Cswy, Miami, FL 33149 USA

**Keywords:** Microbial ecology, Microbial communities, Microbial biooceanography

Correction to: *Nature Communications* 10.1038/s41467-022-30139-4, published online 04 May 2022.

In this article the affiliation details for authors Chase C. James, Lisa A. Zeigler, Robert H. Lampe, Ariel Rabines, Anne Schulberg, Hong Zheng, Andrew E. Allen were incorrectly given as ‘Section of Ecology, Behavior and Evolution, University of California San Diego, 9500 Gilman Dr, La Jolla, CA 92093, United States’ but should have been ‘J. Craig Venter Institute, 4120 Capricorn Lane, La Jolla, CA 92037, United States’ and the affiliation details for author Andrew D. Barton was incorrectly given as ‘J. Craig Venter Institute, 4120 Capricorn Lane, La Jolla, CA 92037, United States’ but should have been ‘Section of Ecology, Behavior and Evolution, University of California San Diego, 9500 Gilman Dr, La Jolla, CA 92093, United States’. The original article has been corrected.

