# Supplementary material for: Influence of nutrient supply on plankton microbiome biodiversity and distribution in a coastal upwelling region
Source: Nat Commun. 2022 May 4;13:2448. doi: 10.1038/s41467-022-30139-4 (PMC9068609; doi:10.1038/s41467-022-30139-4)
Supplement: Supplementary file 1 — Supplementary Information: Influence of nutrient supply on plankton microbiome biodiversity and distribution in a coastal upwelling region [file 41467_2022_30139_MOESM1_ESM.pdf]

Supplementary Information

Influence of nutrient supply on plankton microbiome biodiversity and distribution in a coastal upwelling region

Supplementary Table 1: List of groups used in analysis. Groups listed in bold and shaded in grey are the key functional groups used for the main analysis. Finer taxonomic groups are listed below the broad groups.

Group	# of ASVs	Amplicon Region
**Archaea**	621	16S
**Heterotrophic Bacteria**	17142	16S
**Cyanobacteria**	511	16S
**Photosynthetic Eukaryotic Protists**	7770	18S
**Heterotrophic Eukaryotic Protists**	24311	18S
Prochlorococcus	224	16S
Synechococcus	40	16S
Flavobacteriales	1559	16S
Rhodobacterales	476	16S
SAR 11 Clade	873	16S
Diatoms	620	18S
Dinoflagellates (without Syndiniales)	4494	18S
Syndiniales	5698	18S
Haptophytes	483	18S
Chlorophytes	1056	18S
Metazoans	1943	18S

Supplementary Table 2: Composition of 18Sv9 ASVs endemic to each dataset (columns 2-4) or found in multiple datasets (columns 5-8). Zeros represent groups where no ASVs are found within a given dataset or dataset overlap.

Division	NCOG	Tara Oceans	Tara Polar	NCOG + Tara Oceans	NCOG + Tara Polar	Tara Oceans + Tara Polar	All Datasets
Alveolata	3	12	1	4	0	2	3
Apicomplexa	128	1631	277	136	4	121	27
Apusomonadidae	2	26	25	3	0	5	2
Breviatea	0	3	11	0	0	3	1
Centroheliozoa	8	78	33	15	0	1	8
Cercozoa	104	988	682	191	25	99	120
Chlorophyta	528	2407	248	419	12	66	77
Choanoflagellida	19	136	76	25	4	5	29
Ciliophora	349	3447	1155	589	46	572	300
Conosa	0	26	22	0	0	2	2
Cryptophyta	8	145	72	18	1	7	15
Dinoflagellata	3491	21790	6626	5617	124	977	1993
Discoba	543	8304	9934	172	11	294	224
Foraminifera	13	949	265	14	2	48	8
Fungi	52	1145	403	46	7	97	65
Haptophyta	98	559	153	269	4	32	109
Katablepharidophyta	3	43	21	6	0	2	6
Lobosa	27	451	142	20	1	33	11
Mesomycetozoa	3	24	3	4	0	6	3
Metazoa	1113	26431	3103	560	41	274	188
Ochrophyta	420	5769	2211	528	30	161	288
Opalozoa	107	685	147	147	12	38	90
Opisthokonta	1	51	1	13	0	2	4
Picozoa	12	197	71	7	1	4	17
Prasinodermophyta	2	1	0	3	1	0	2
Pseudofungi	40	329	197	40	2	16	30
Radiolaria	324	11290	956	869	16	129	338
Rhodophyta	23	347	8	40	0	9	11
Sagenista	46	663	204	106	6	40	66
Stramenopiles	0	16	9	1	0	2	4
Streptophyta	46	249	79	28	1	11	9
Telonemia	24	189	86	39	2	11	46
Eukaryota	1	5	3	2	0	0	0
Hilomonadea	2	10	7	3	0	0	0
Mantamonadidea	0	2	0	0	0	0	0
Other	7350	93539	25186	3202	133	4637	947
Total	14890	181937	52417	13136	486	7706	5043

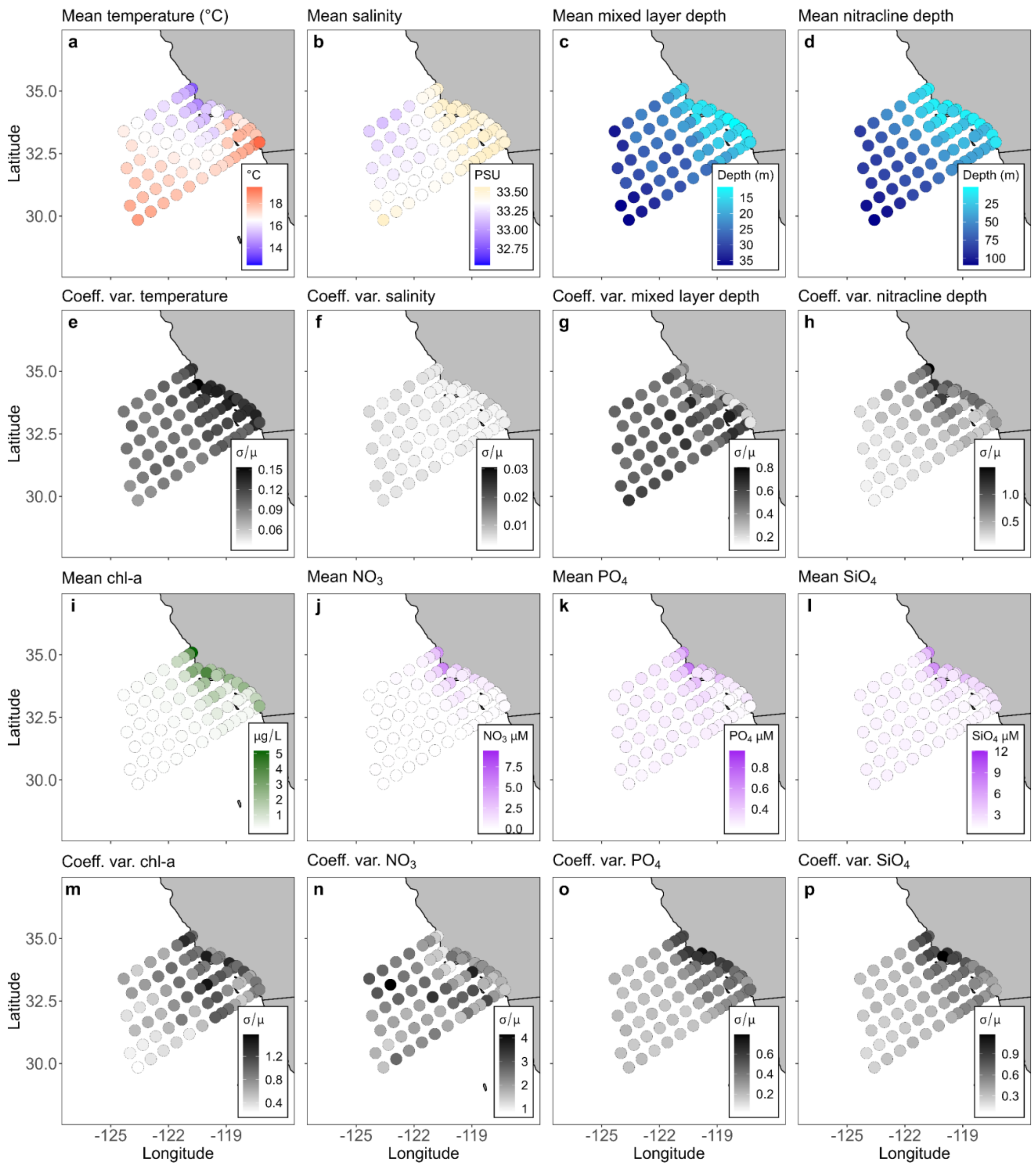

Supplementary Fig. 1: Mean spatial gradients in physical and ecological variables shown in color: (**a**) temperature (°C), (**b**) salinity (PSU), (**c**) mixed layer depth (m), (**d**) nitracline depth (m), (**i**) chlorophyll *a* (µg/L), (**j**) nitrate (µM), (**k**) phosphate (µM), and (**l**) silicate (µM). Spatial gradients in the coefficient of variation (Coeff. var.) are shown in grayscale for: (**e**) temperature, (**f**) salinity, (**g**) mixed layer depth, (**h**) nitracline depth, (**m**) chlorophyll *a*, (**n**) nitrate, (**o**) phosphate, and (**p**) silicate.

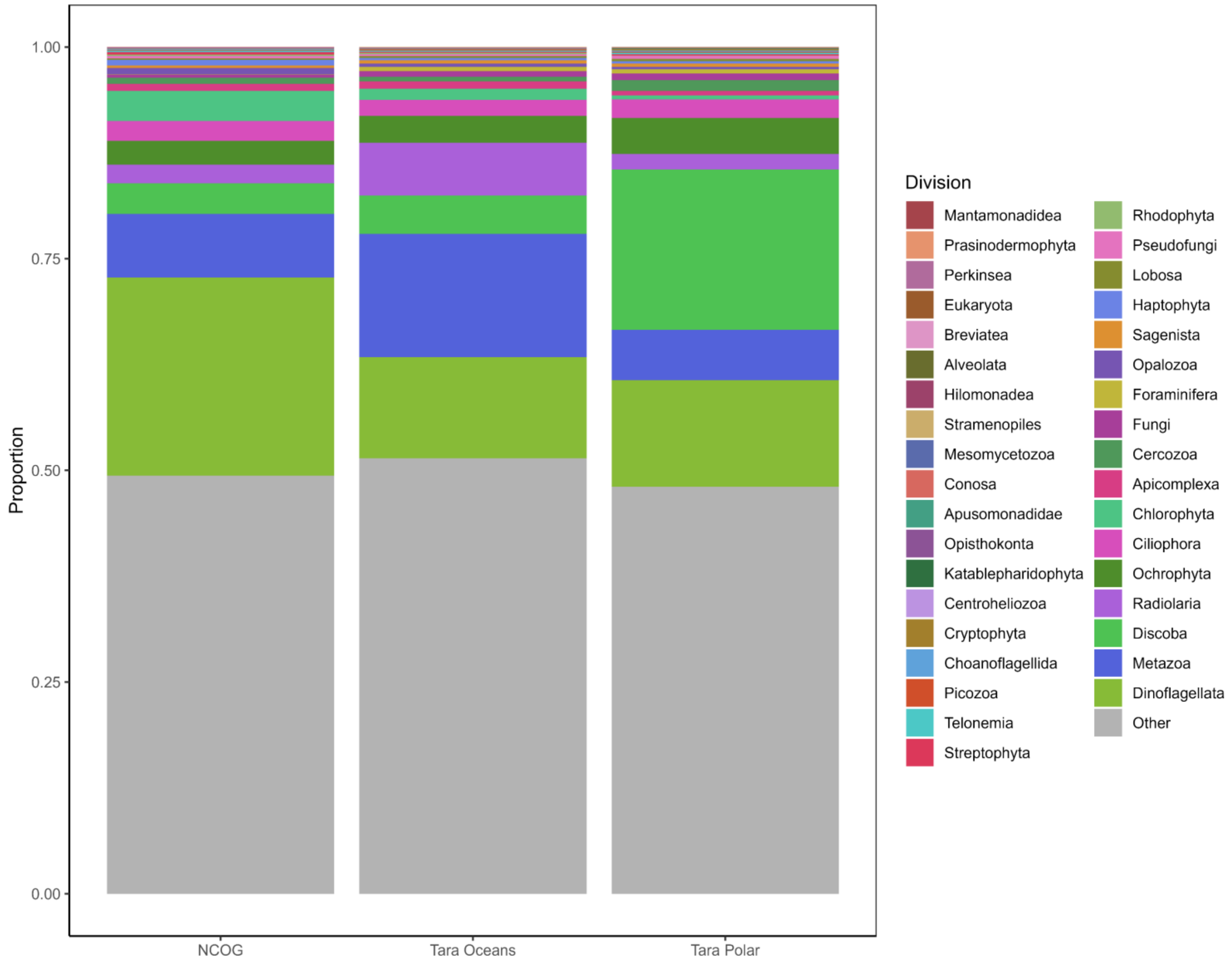

Supplementary Fig. 2: Proportional taxonomic composition of ASVs that are endemic to NCOG, TARA Oceans, and TARA Polar. Colors represent the proportional dominance of broad taxonomic groups within the ASVs that are endemic to each dataset. Total number of endemic ASVs per dataset can be found in Fig. 1e.

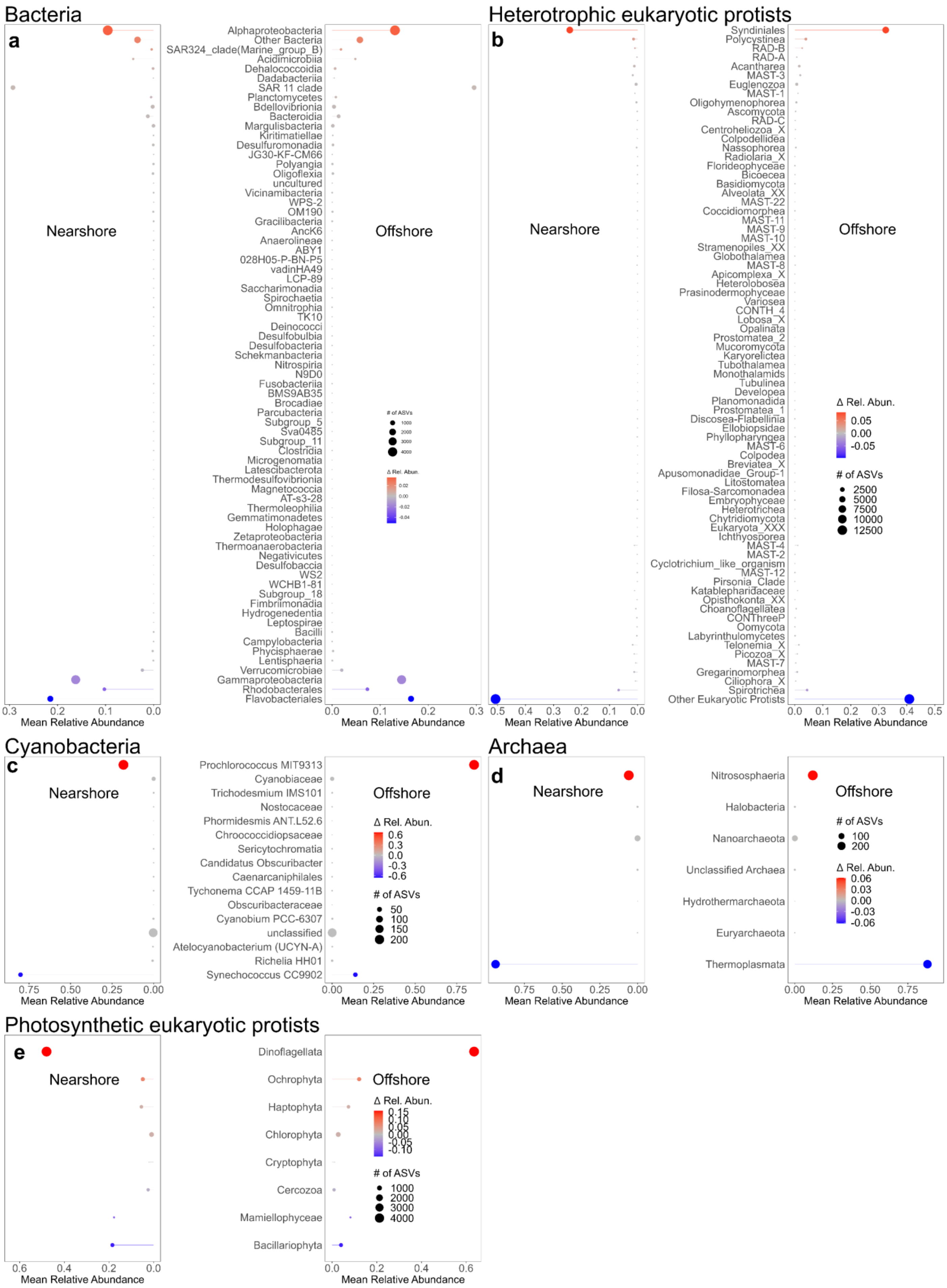
Bacteria
a
Nearshore
Offshore
Alphaproteobacteria
Other Bacteria
SAR324_clade(Marine_group_B)
Acidimicrobiia
Dehalococcoidia
Dadabacteriia
SAR 11 clade
Planctomycetes
Bdellovibrionia
Bacteroidia
Margulisbacteria
Kiritimatiellae
Desulfuromonadia
JG30-KF-CM66
Polyangia
Oligoflexia
uncultured
Vicinamibacteria
WPS-2
OM190
Gracilibacteria
AncK6
Anaerolineae
ABY1
028H05-P-BN-P5
vadinHA49
LCP-89
Saccharimonadia
Spirochaetia
Omnitrophia
TK10
Deinococci
Desulfobulbia
Desulfobacteria
Schekmanbacteria
Nitrospiria
N9D0
Fusobacteriia
BMS9AB35
Brocadiae
Parcubacteria
Subgroup_5
Sva0485
Subgroup_11
Clostridia
Microgenomatia
Latescibacterota
Thermodesulfovibrionia
Magnetococcia
AT-s3-28
Thermoleophilia
Gemmatimonadetes
Holophagae
Zetaproteobacteria
Thermoanaerobacteria
Negativicutes
Desulfobaccia
WS2
WCHB1-81
Subgroup_18
Fimbriimonadia
Hydrogenedentia
Leptospirae
Bacilli
Campylobacteria
Phycisphaerae
Lentisphaeria
Verrucomicrobiae
Gammaproteobacteria
Rhodobacterales
Flavobacteriales
0.3 0.2 0.1 0.0
0.0 0.1 0.2 0.3
Mean Relative Abundance
Heterotrophic eukaryotic protists
b
Nearshore
Offshore
Syndiniales
Polycystinea
RAD-B
RAD-A
Acantharea
MAST-3
Euglenozoa
MAST-1
Oligohymenophorea
Ascomycota
RAD-C
Centroheliozoa_X
Colpodellidea
Nassophorea
Radiolaria_X
Florideophyceae
Bicoecea
Basidiomycota
Alveolata_XX
MAST-22
Coccidiomorphea
MAST-11
MAST-9
MAST-10
Stramenopiles_XX
Globothalamea
MAST-8
Apicomplexa_X
Heterolobosea
Prasinodermophyceae
Variosea
CONTH_4
Lobosa_X
Opalinata
Prostomatea_2
Mucoromycota
Karyorelictea
Tubothalamea
Monothalamids
Tubulinea
Developea
Planomonadida
Prostomatea_1
Discosea-Flabellinia
Ellobiopsidae
Phyllopharyngea
MAST-6
Colpodea
Breviatea_X
Apusomonadidae_Group-1
Litostomatea
Filosa-Sarcomonadea
Embryophyceae
Heterotrichea
Chytridiomycota
Eukaryota_XXX
Ichthyosporea
MAST-4
MAST-2
Cyclotrichium_like_organism
MAST-12
Pirsonia_Clade
Katablepharidaceae
Opisthokonta_XX
Choanoflagellatea
CONThreeP
Oomycota
Labyrinthulomycetes
Telonemia_X
Picozoa_X
MAST-7
Gregarinomorphea
Ciliophora_X
Spirotrichea
Other Eukaryotic Protists
Δ Rel. Abun.
0.05
0.00
-0.05
# of ASVs
2500
5000
7500
10000
12500
0.5 0.4 0.3 0.2 0.1 0.0
0.0 0.1 0.2 0.3 0.4 0.5
Mean Relative Abundance
Cyanobacteria
c
Nearshore
Offshore
Prochlorococcus MIT9313
Cyanobiaceae
Trichodesmium IMS101
Nostocaceae
Phormidesmis ANT.L52.6
Chroococcidiopsaceae
Sericytochromatia
Candidatus Obscuribacter
Caenarcaniphilales
Tychonema CCAP 1459-11B
Obscuribacteraceae
Cyanobium PCC-6307
unclassified
Atelocyanobacterium (UCYN-A)
Richelia HH01
Synechococcus CC9902
Δ Rel. Abun.
0.6
0.3
0.0
-0.3
-0.6
# of ASVs
50
100
150
200
0.75 0.50 0.25 0.00
0.00 0.25 0.50 0.75
Mean Relative Abundance
Archaea
d
Nearshore
Offshore
Nitrososphaeria
Halobacteria
Nanoarchaeota
Unclassified Archaea
Hydrothermarchaeota
Euryarchaeota
Thermoplasmata
# of ASVs
100
200
Δ Rel. Abun.
0.06
0.03
0.00
-0.03
-0.06
0.75 0.50 0.25 0.00
0.00 0.25 0.50 0.75
Mean Relative Abundance
Photosynthetic eukaryotic protists
e
Nearshore
Offshore
Dinoflagellata
Ochrophyta
Haptophyta
Chlorophyta
Cryptophyta
Cercozoa
Mamiellophyceae
Bacillariophyta
Δ Rel. Abun.
0.15
0.10
0.05
0.00
-0.05
-0.10
# of ASVs
1000
2000
3000
4000
0.6 0.4 0.2 0.0
0.0 0.2 0.4 0.6
Mean Relative Abundance

Supplementary Fig. 3: Mean relative abundance of taxonomic groups in both the nearshore and offshore clusters for major groups: (**a**) bacteria, (**b**) heterotrophic eukaryotic protists, (**c**) cyanobacteria, (**d**) archaea, and (**e**) photosynthetic eukaryotic protists. Mean relative abundance is calculated as the mean abundance of all ASVs within a taxonomic group per cluster. Taxonomic groups are ordered, from top to bottom, by their difference in mean relative abundance within the offshore versus the nearshore: Δ Mean Relative Abundance = Mean Offshore Relative Abundance – Mean Nearshore Relative Abundance. A positive difference (red) indicates the mean relative abundance is greater in the offshore and a negative difference (blue) indicates the mean relative abundance is greater in the nearshore. The size of the circles represents the number of ASVs found within each taxonomic group. Listed taxonomic groups include those that were most abundant or are thought to be ecologically important. The rest of the ASVs are included in the "Other" categories found in each subplot.

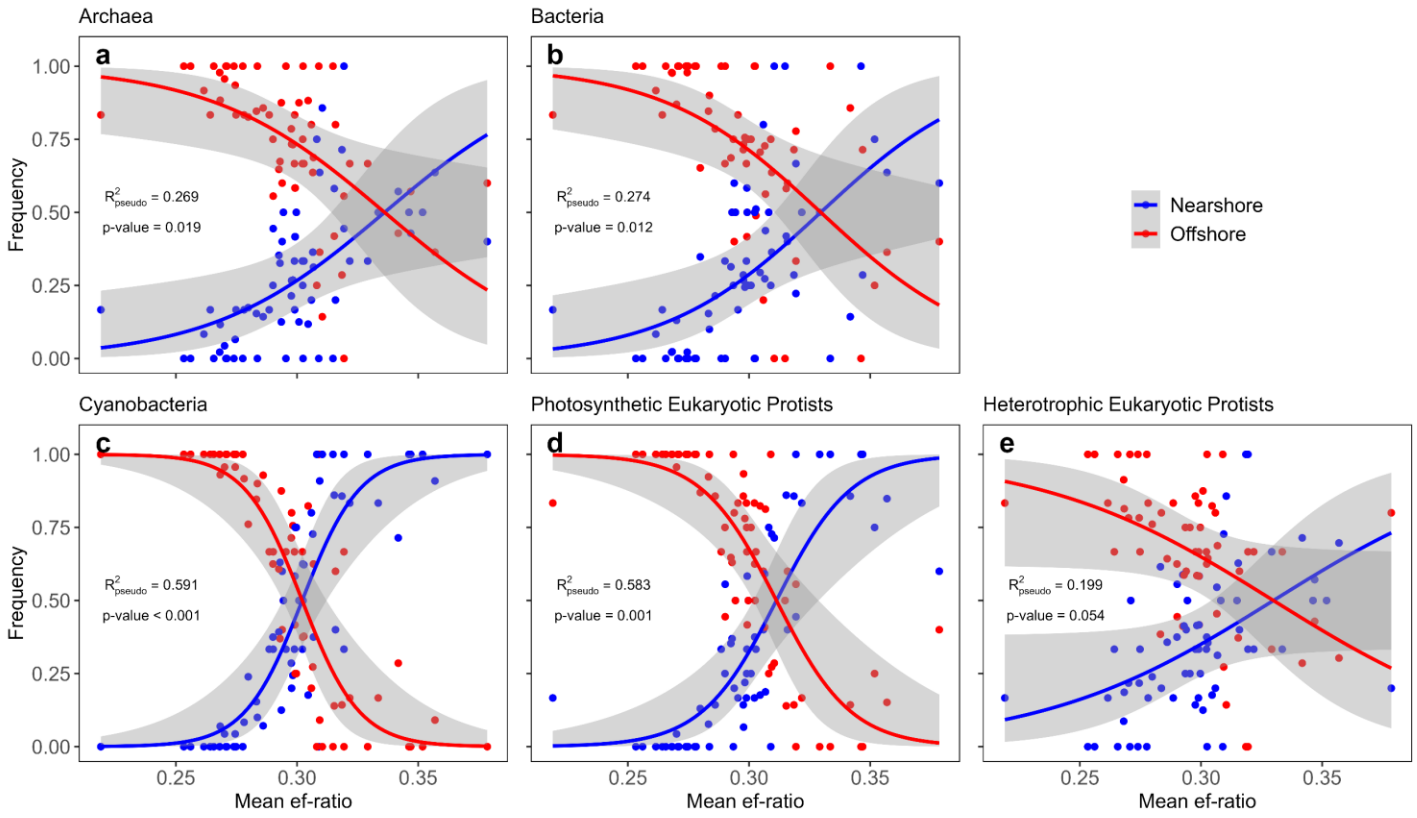

Supplementary Fig. 4: Relationship between ef-ratio (calculated from Eq. 2 of Laws et al. 2011) and the frequency of SOM clusters per station from 2014-2020 for the five major taxonomic groups: (**a**) archaea, (**b**) bacteria, (**c**) cyanobacteria, (**d**) photosynthetic eukaryotic protists, and (**e**) heterotrophic eukaryotic protists. Cragg and Uhler's pseudo R^2 was used to assess the goodness of fit between mean ef-ratio and frequency. Shading represents the 95% confidence interval around each model fit.

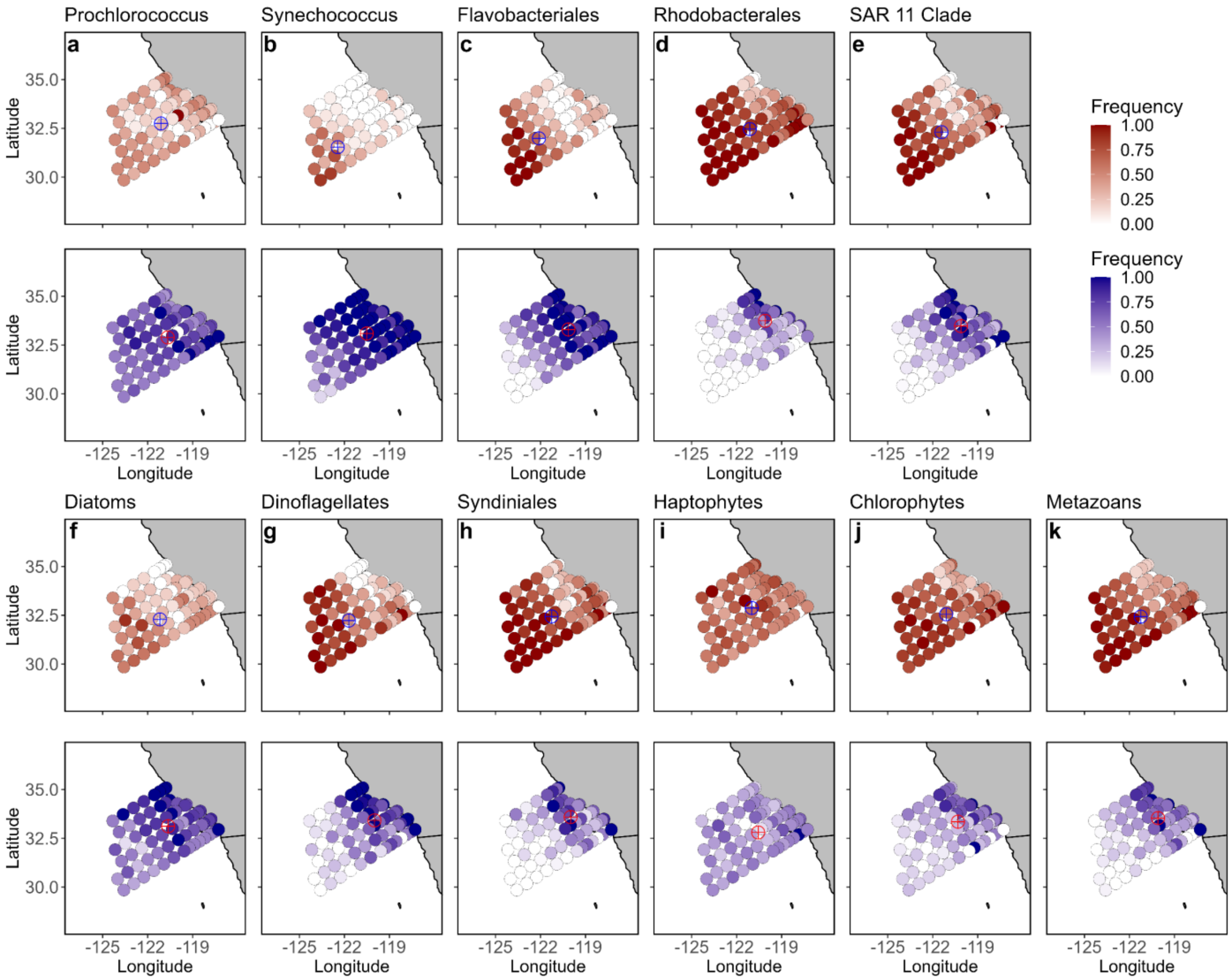

Supplementary Fig. 5: Nearshore and offshore gradients in community structure within the eleven taxonomic groups: (**a**) Prochlorococcus, (**b**) Synechococcus, (**c**) Flavobacteriales, (**d**) Rhodobacteriales, (**e**) SAR 11 Clade, (**f**) Diatoms, (**g**) Dinoflagellates, (**h**) Syndiniales, (**i**) Haptophytes, (**j**) Chlorophytes, and (**k**) Metazoans. Colors indicate the frequency that the community at each location is offshore (red, top row) or nearshore (blue, bottom row) in character. The designation of nearshore vs. offshore community is determined by the cluster whose weighted centroid is closer to the coast. The weighted centroid for each cluster is shown as a target in the opposite color.

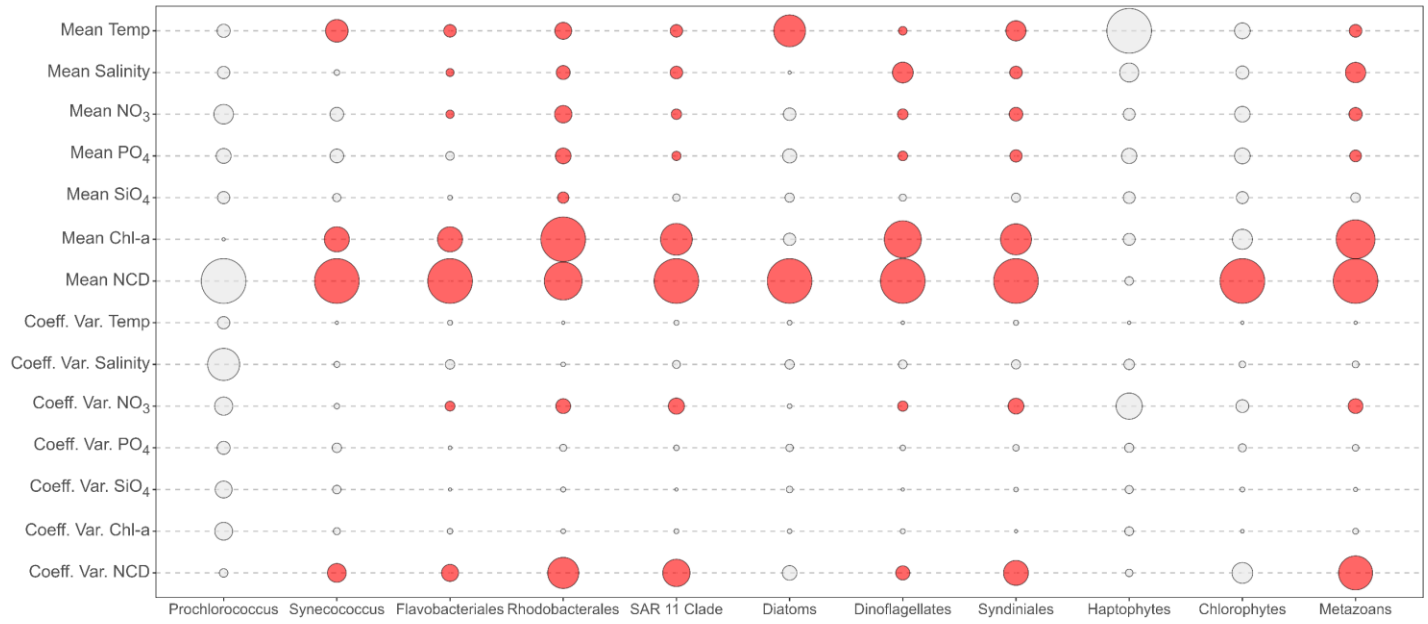

Supplementary Fig. 6: Relative importance of all explanatory variables (mean and coefficient of variation) used to predict the frequency of offshore vs. nearshore clusters at a given station for taxonomic specific groups. Relationships were assessed via a generalized linear model with a binomial fit. Larger circles represent lower AIC values within a column. Relationships that are not significant ($p > 0.05$) are colored grey. Circles and their associated AIC values should not be compared across columns. Relationships were analyzed between the frequency of observed community clusters and the mean and coefficient of variation (Coeff. Var.) of environmental variables. Environmental variables included: temperature (Temp), salinity, NO_3, PO_4, SiO_4, chlorophyll a (Chl-a), and nitracline depth (NCD).

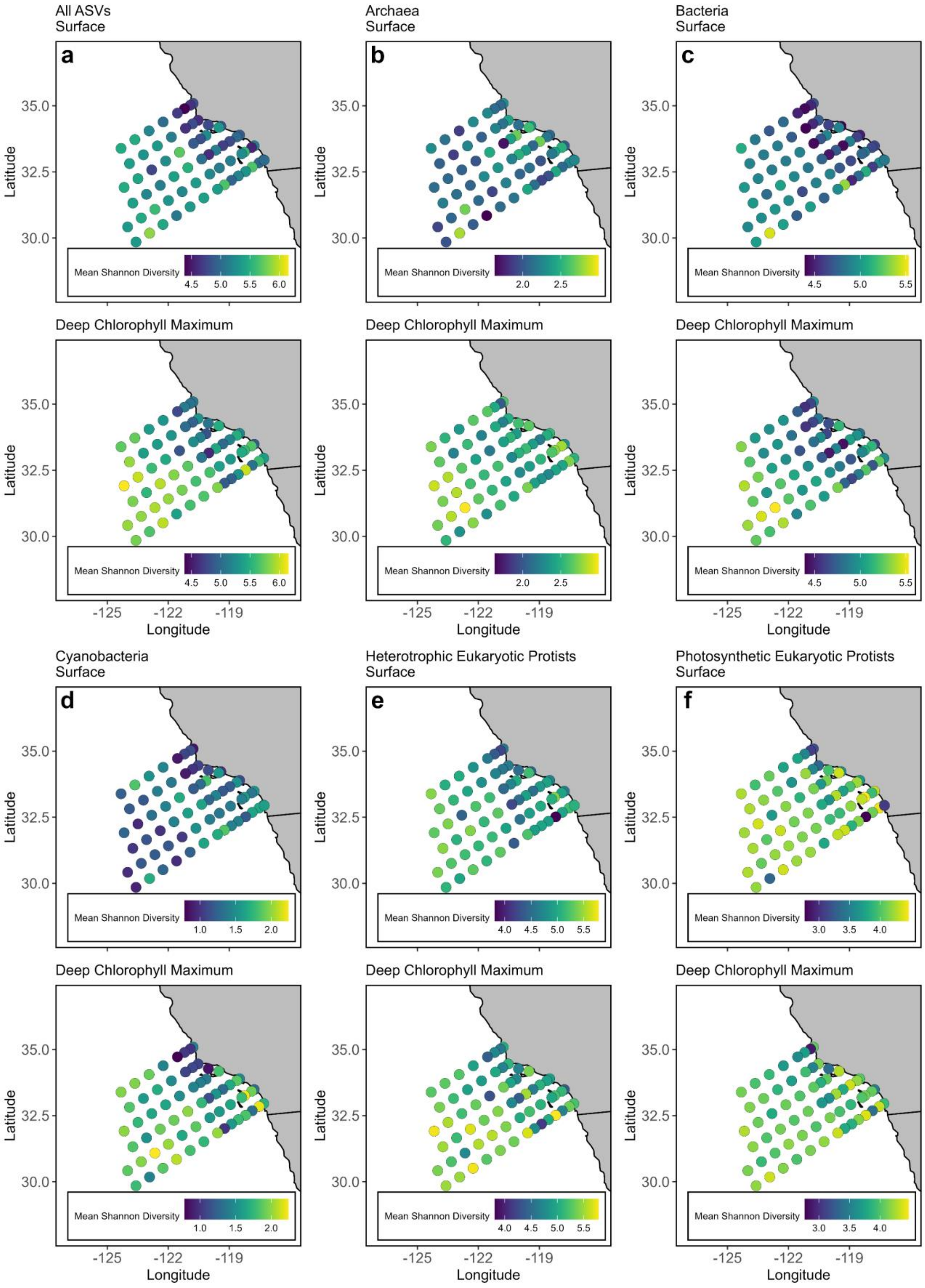

Supplementary Fig. 7: Mean alpha diversity for (**a**) all ASVs and the five major groups: (**b**) archaea, (**c**) bacteria, (**d**) cyanobacteria, (**e**) heterotrophic eukaryotic protists, and (**f**) photosynthetic eukaryotic protists at each CalCOFI station in both surface samples (top panel) and deep chlorophyll maximum samples (bottom panel) across all years (2014-2020). Scale bars for each group are consistent across depths (surface and deep chlorophyll maximum).

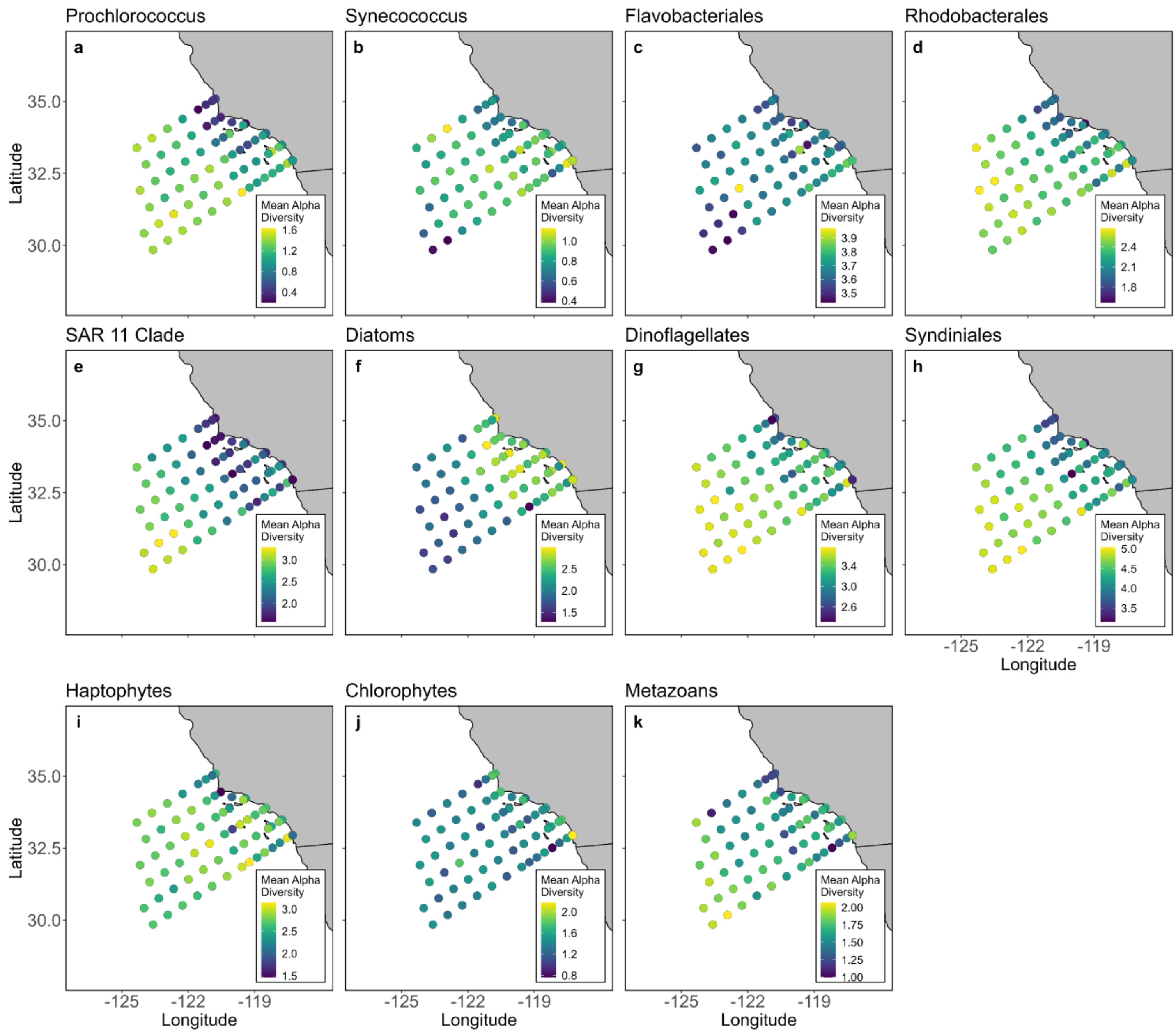

Supplementary Fig. 8: Mean alpha diversity for the eleven taxonomic groups: (**a**) Prochlorococcus, (**b**) Synechococcus, (**c**) Flavobacteriales, (**d**) Rhodobacteriales, (**e**) SAR 11 Clade, (**f**) Diatoms, (**g**) Dinoflagellates, (**h**) Syndiniales, (**i**) Haptophytes, (**j**) Chlorophytes, and (**k**) Metazoans at each CalCOFI station across all samples and years (2014-2020).

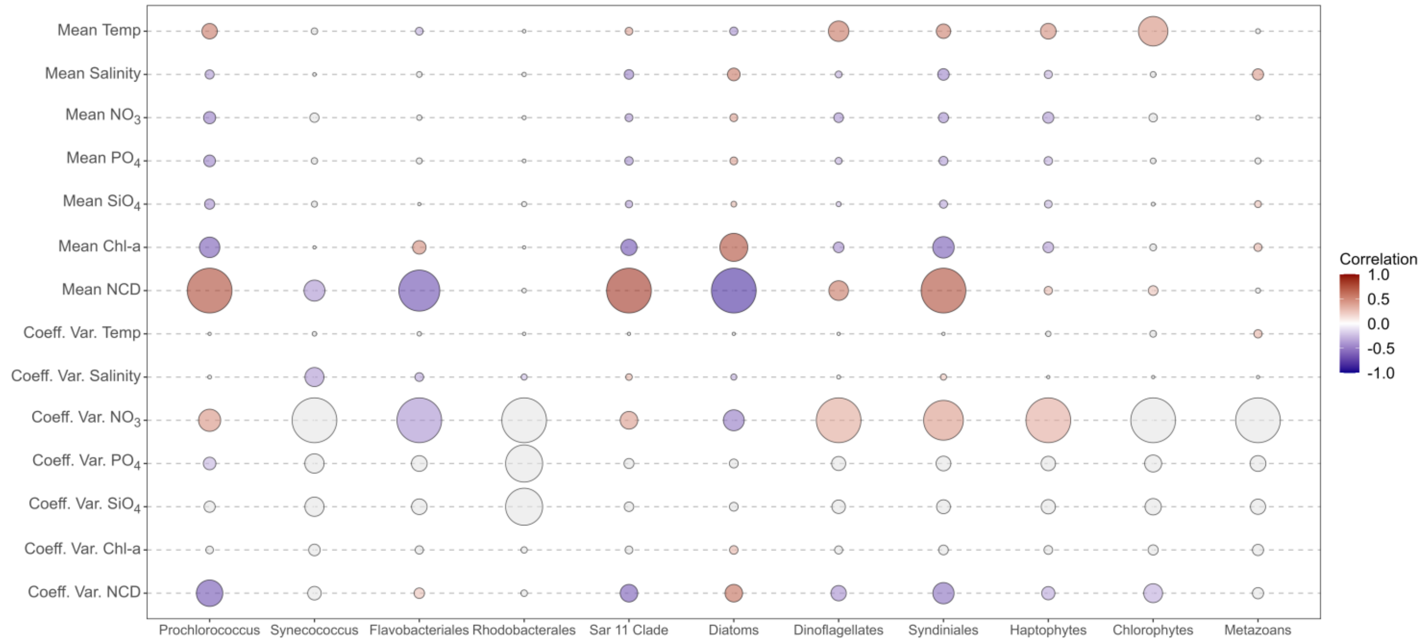

Supplementary Fig. 9: Relative importance of all explanatory variables (mean and coefficient of variation) as used to predict mean alpha diversity at a given station for the finer taxonomic groups. Relationships were assessed via a generalized linear model with a gaussian fit. Larger circles represent lower AIC values within a column. Circles and their associated AIC values should not be compared across columns. Color represents the correlation coefficient between each explanatory variable and mean alpha diversity. Gray circles represent relationships that are not significant ($p > 0.05$). Shannon index was used as the primary measure of diversity and was calculated as the mean per station per cruise for this analysis. Relationships between environmental variables and diversity were assessed via a generalized linear model with a gaussian fit. Larger circles represent lower AIC values within a column. Circles and their associated AIC values should not be compared across columns. Color represents the correlation coefficient between each explanatory variable and mean alpha diversity. Gray circles represent relationships that are not significant ($p > 0.05$). Relationships were analyzed between diversity and the mean and coefficient of variation (Coeff. Var.) of environmental variables. Environmental variables included: temperature (Temp), salinity, NO_3, PO_4, SiO_4, chlorophyll a (Chl-a), and nitracline depth (NCD).

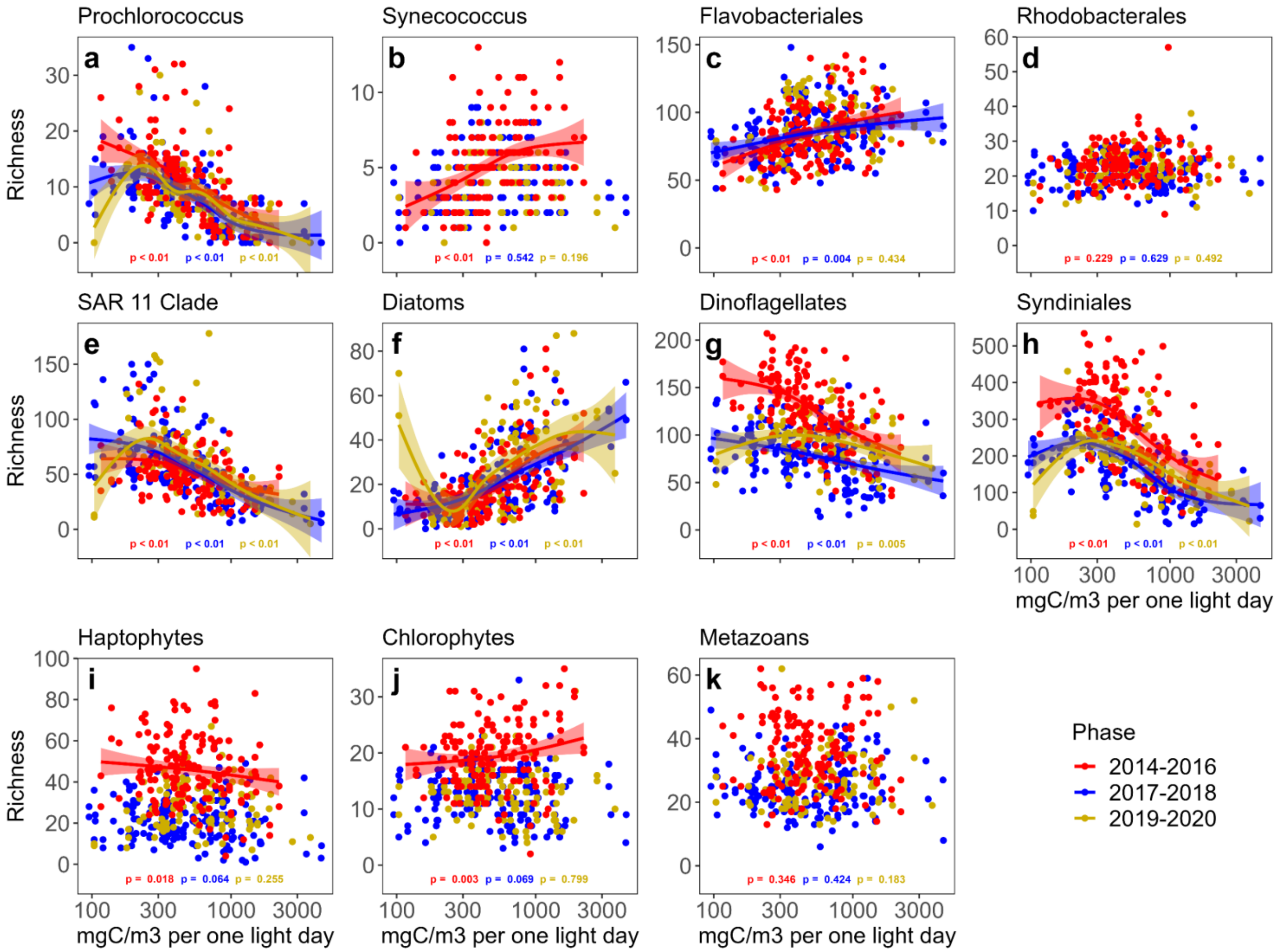

Supplementary Fig. 10: Productivity-diversity relationship for all eleven taxonomic groups: (**a**) Prochlorococcus, (**b**) Synechococcus, (**c**) Flavobacteriales, (**d**) Rhodobacterales, (**e**) SAR 11 Clade, (**f**) Diatoms, (**g**) Dinoflagellates, (**h**) Syndiniales, (**i**) Haptophytes, (**j**) Chlorophytes, and (**k**) Metazoans. The data was subset to include only productivity station samples where ^{14}C was measured. Productivity-diversity relationships were fit with a generalized additive model (GAM). Significant relationships are denoted by a red line (2014-2016), blue line (2017-2018), or gold line (2019-2020). Lines represent the best GAM fit with shading representing a 99% confidence interval. Richness (total number of ASVs) rather than Shannon Index is used in this figure as this is the standard for fitting productivity-diversity relationships[45,46].

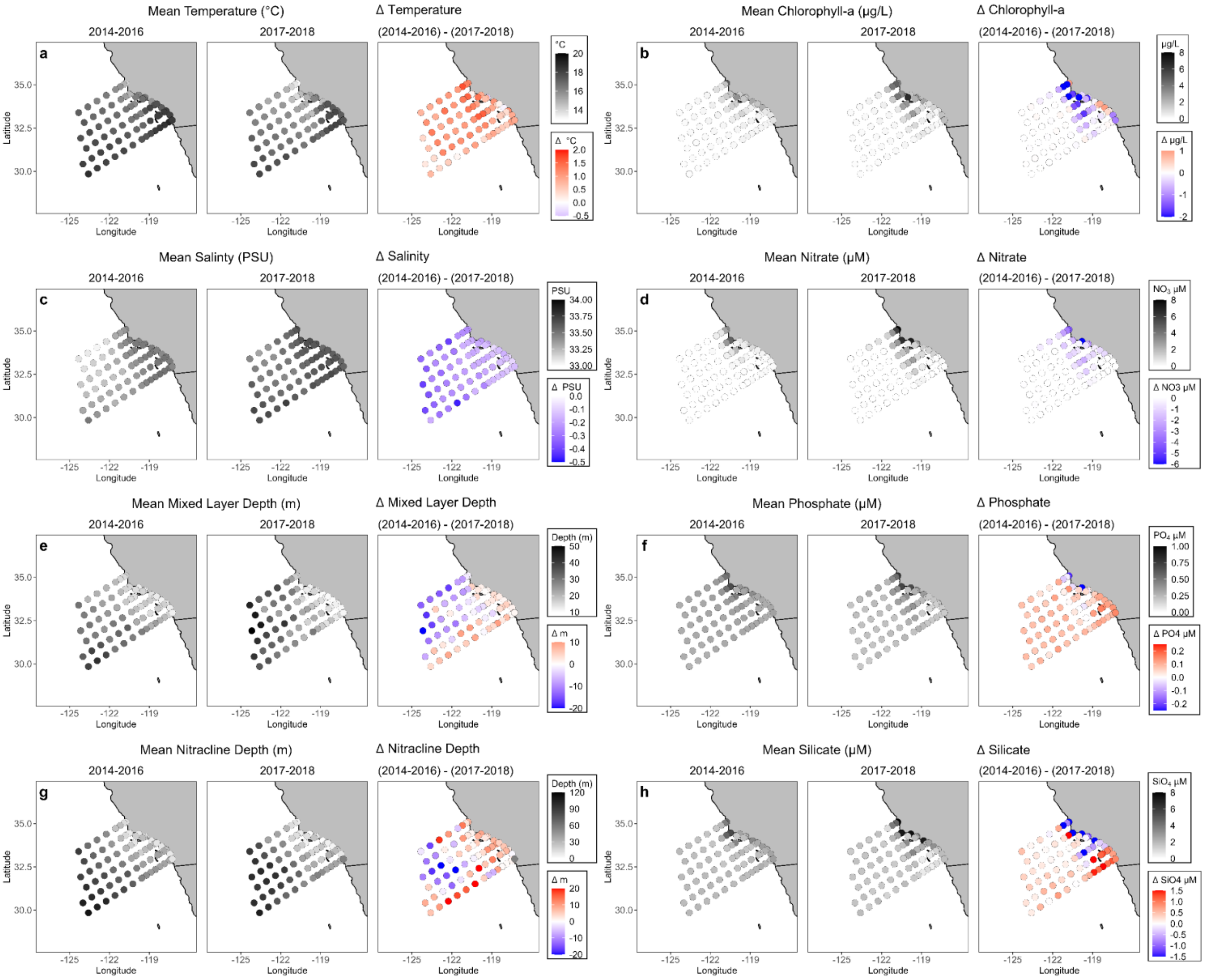

Supplementary Fig. 11: Mean spatial gradients of physical and ecological variables in 2014-2016 and 2017-2018 are shown in grayscale. The difference between the two time periods is shown in color. Variables include: (**a**) temperature (°C), (**b**) salinity (PSU, practical salinity units), (**c**) mixed layer depth (m), (**d**) nitracline depth (m), (**e**) chlorophyll *a* (µg/L), (**f**) nitrate (µM), (**g**) phosphate (µM), and (**h**) silicate (µM)

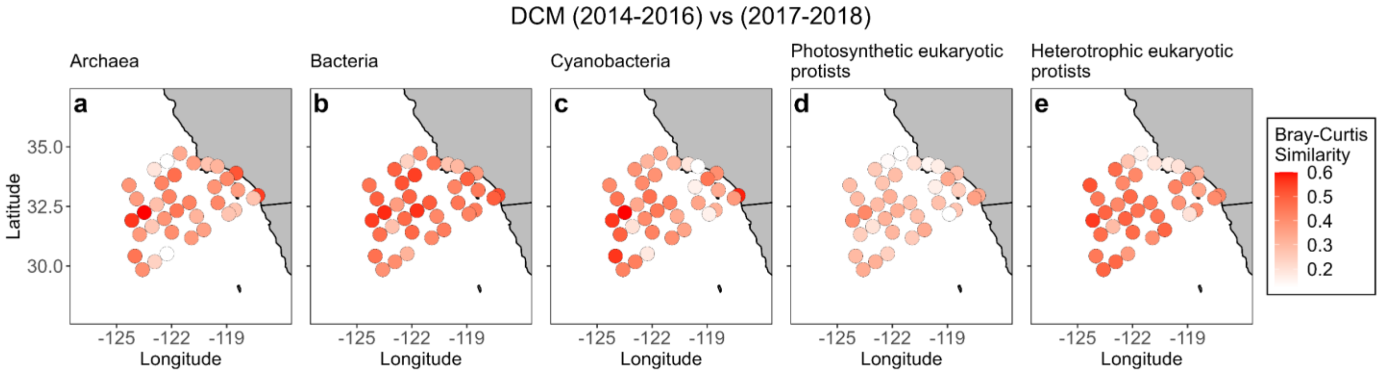

Supplementary Fig. 12: Maps of the mean Bray-Curtis similarity between deep chlorophyll maximum (DCM) samples from the warm (2014-2016) and cool (2017-2018) phase for each station for our five main groups: (**a**) archaea, (**b**) bacteria, (**c**) cyanobacteria, (**d**) photosynthetic eukaryotic protists, and (**e**) heterotrophic eukaryotic protists.

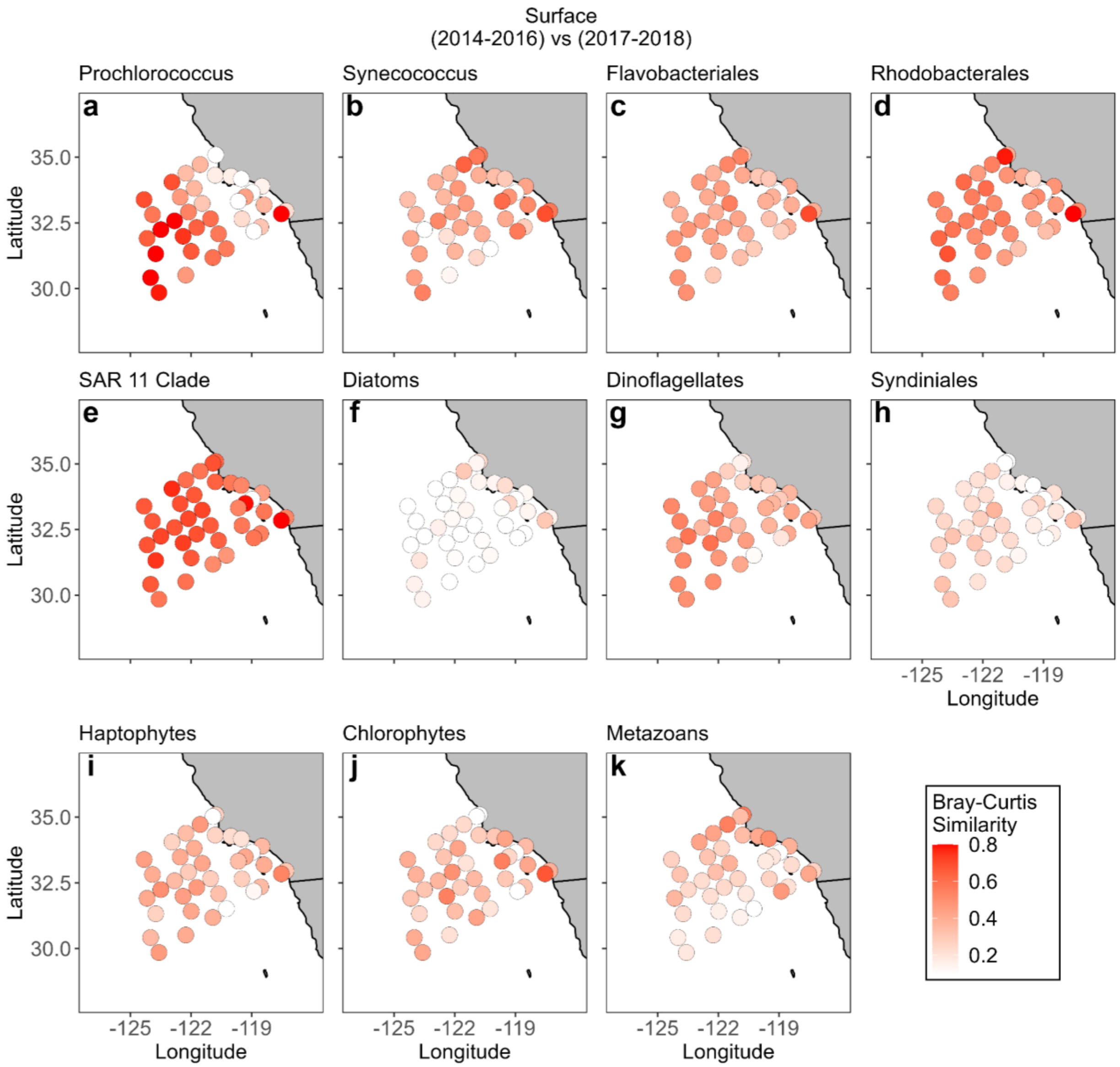

Supplementary Fig. 13: Maps of the mean Bray-Curtis similarity between surface samples from the warm (2014-2016) and cool (2017-2018) phase for each station. Maps show surface samples for our eleven taxonomic groups: (**a**) Prochlorococcus, (**b**) Synechococcus, (**c**) Flavobacteriales, (**d**) Rhodobacteriales, (**e**) SAR 11 Clade, (**f**) Diatoms, (**g**) Dinoflagellates, (**h**) Syndiniales, (**i**) Haptophytes, (**j**) Chlorophytes, and (**k**) Metazoans.

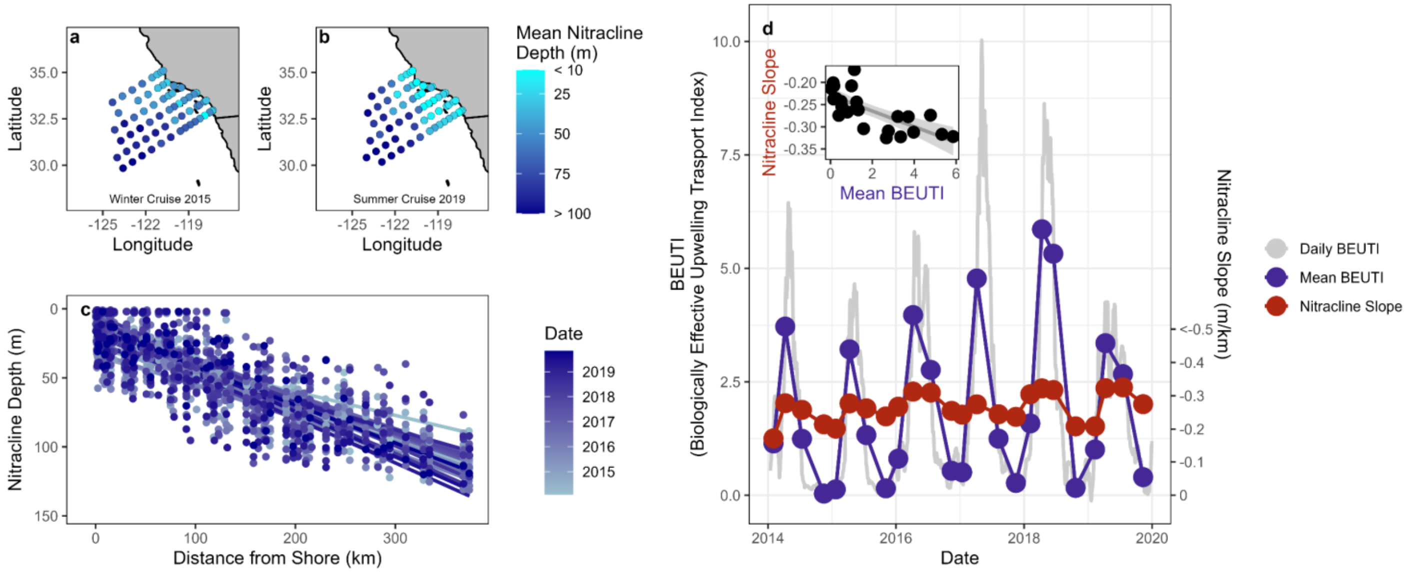

Supplementary Fig. 14: Examples of cruises with variable regional nitracline slopes. (**a**), winter 2015 where there was a shallow regional nitracline slope (similar nitracline depth across the entire region). (**b**), summer 2019 where there was strong nearshore upwelling (shallow nitracline) contrasted with a deep nitracline far offshore (**c**), Regional slope of the nitracline for each cruise. Color of the points and lines represents the mean date for a given cruise. Slopes were fit with a generalized linear model (**d**), Relationship between Biological Effective Upwelling Transport Index (BEUTI) and the regional nitracline slope. Grey line shows the daily BEUTI values while the blue line shows a 3-month moving average centered around the mean cruise date (points). Red line shows the nitracline slope for each cruise. The inlaid plot shows the correlation between Mean BEUTI and Nitracline Slope where higher BEUTI values correlated with steeper slopes in the nitracline.

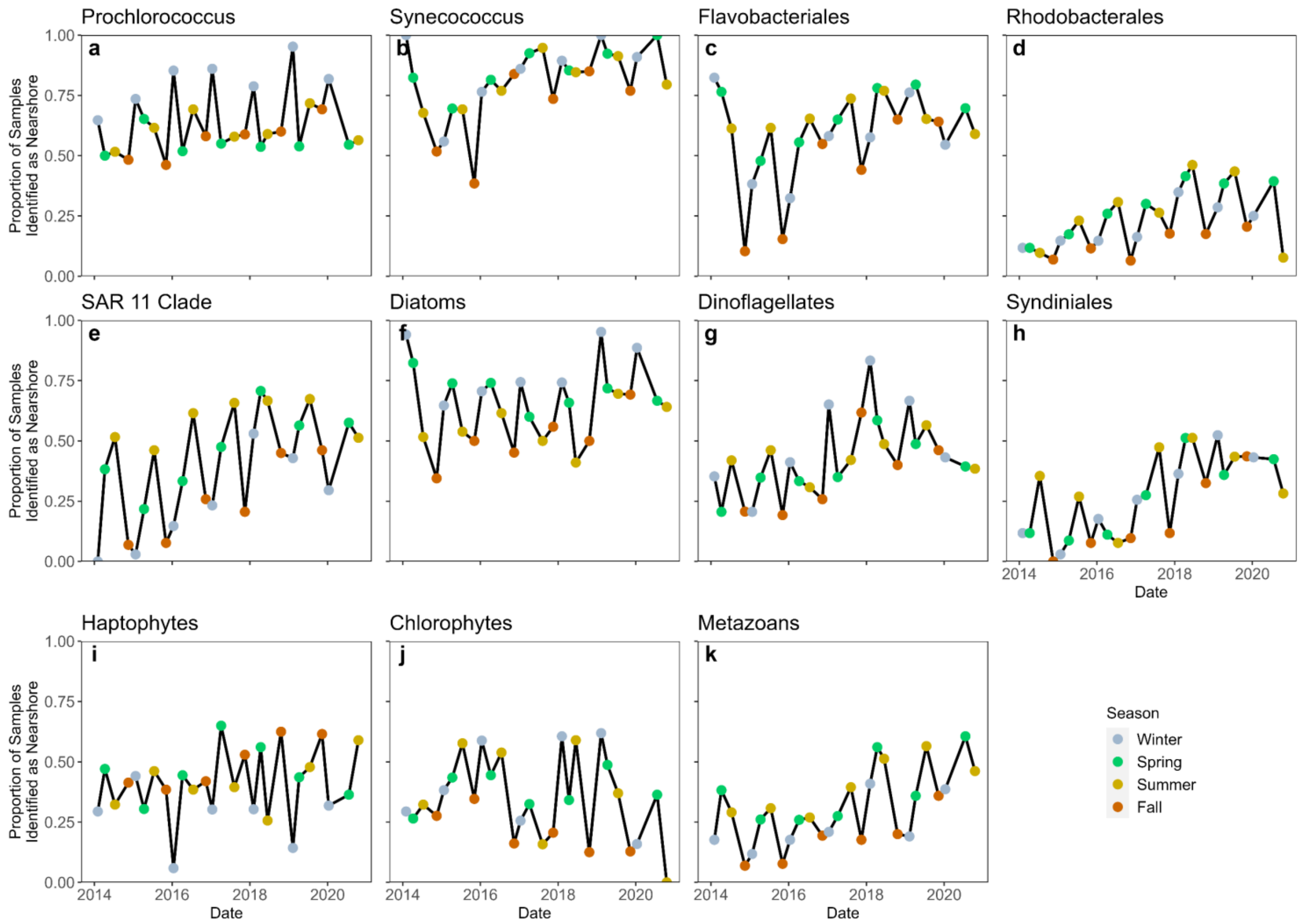

Supplementary Fig. 15: Time series illustrating the proportion of samples per cruise that were identified as “nearshore” communities. Points are colored based on the season during which each cruise took place. Panel represent each of our eleven taxonomic groups: (**a**) Prochlorococcus, (**b**) Synechococcus, (**c**) Flavobacteriales, (**d**) Rhodobacterales, (**e**) SAR 11 Clade, (**f**) Diatoms, (**g**) Dinoflagellates, (**h**) Syndiniales, (**i**) Haptophytes, (**j**) Chlorophytes, and (**k**) Metazoans.

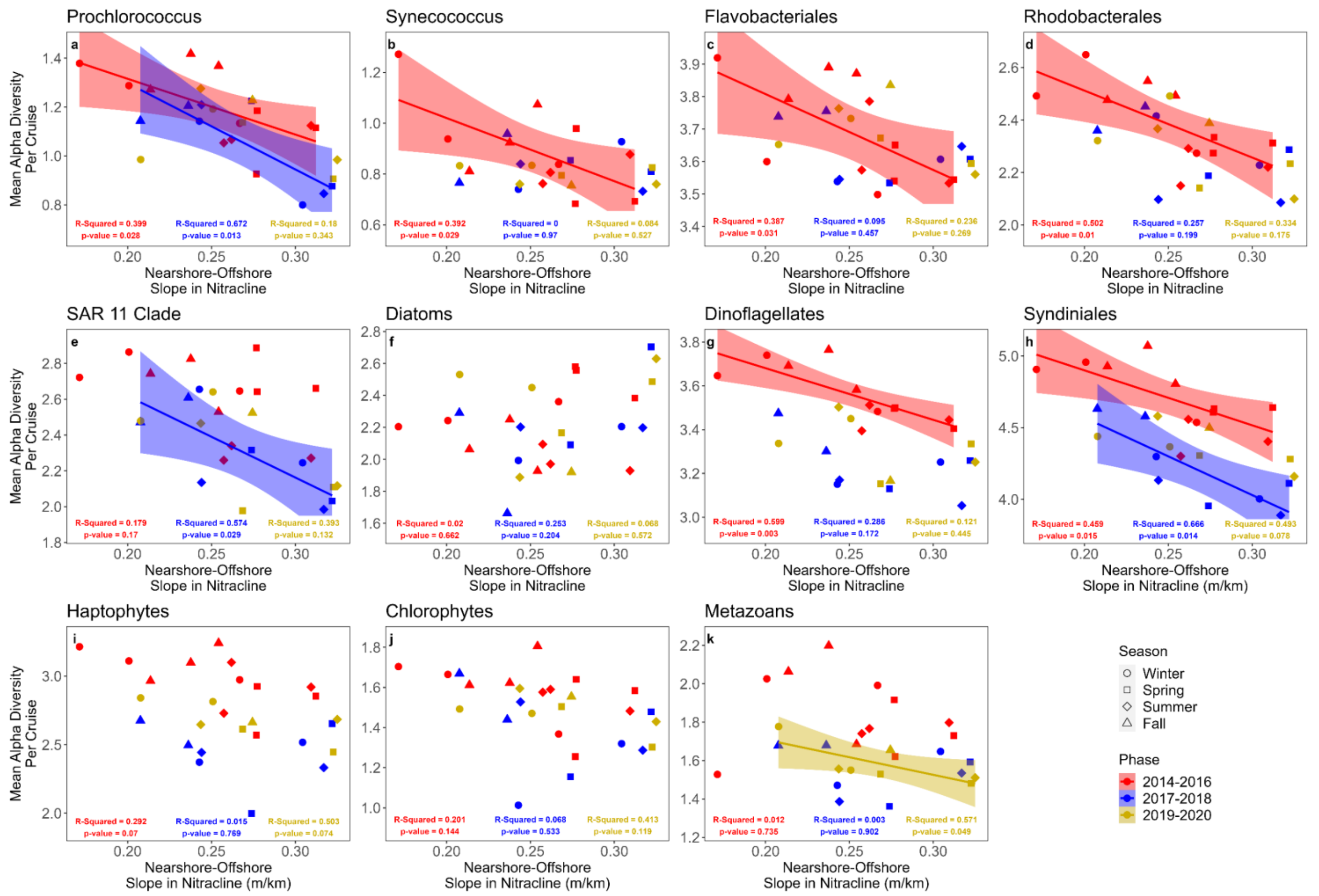

Supplementary Fig. 16: Mean alpha diversity in relation to the slope in the nitracline depth across the entire region. Shapes represent the different seasons during which cruises took place (circle = winter, square = spring, diamond = summer, triangle = fall) and the colors represent samples that were collected from 2014-2016 (red), 2017-2018 (blue), or 2019-2020 (gold). Data were fitted as separate linear models per phase. Shading represents the 95% confidence interval around the model fit. Panels represent each of our eleven taxonomic groups: (**a**) Prochlorococcus, (**b**) Synechococcus, (**c**) Flavobacteriales, (**d**) Rhodobacteriales, (**e**) SAR 11 Clade, (**f**) Diatoms, (**g**) Dinoflagellates, (**h**) Syndiniales, (**i**) Haptophytes, (**j**) Chlorophytes, and (**k**) Metazoans.

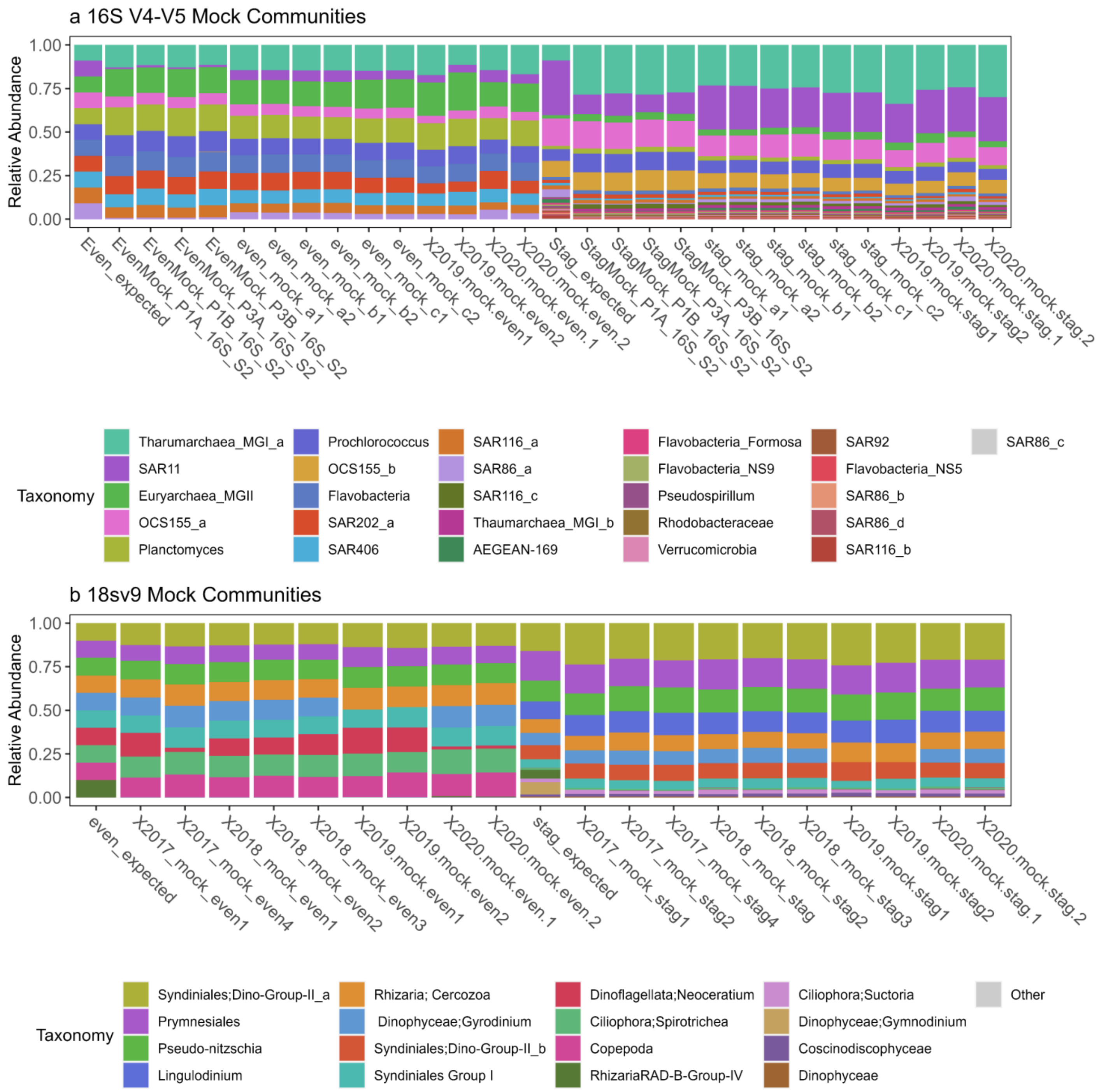

Supplementary Fig. 17: Even and staggered mock communities for: (**a**), 16S and (**b**), 18Sv9. Custom mock communities were the same as those found in Parada et al. 2016[75]. SAR86_c is expected in our staggered community (0.002 expected relative abundance) but is not seen in any mock community samples.
