# Supplementary material for: Influence of nutrient supply on plankton microbiome biodiversity and distribution in a coastal upwelling region
Source: Nat Commun. 2022 May 4;13:2448. doi: 10.1038/s41467-022-30139-4 (PMC9068609; doi:10.1038/s41467-022-30139-4)
Supplement: Supplementary file 3 — Description of Additional Supplementary Files [file 41467_2022_30139_MOESM3_ESM.pdf]

File Name: Supplementary Data 1

Description: For each of the five major taxonomic groups, differences in community structure, as classified by SOM clusters, were driven by the differential relative abundance of ASVs. In this supplementary data file, we highlight those ASVs that show the greatest differential abundance (> 99th percentile) between nearshore and offshore clusters. Full taxonomy, the major taxonomic group they are associated with, the dominant cluster (Nearshore or Offshore), and the delta mean relative abundance between clusters is shown for each ASV.

File Name: Supplementary Data 2

Description: Associated metadata for all samples. Data includes both sample metadata (Name, Cruise ID, Station ID, Date, Time, Latitude, Longitude, Distance from coast, Associated Niskin bottles, Depth, and Volume Filtered) and environmental data (Temperature, Salinity, Oxygen, nutrient concentrations, chlorophyll *a* concentrations, mixed layer depth, nitracline depth, and primary productivity estimates.
