# Supplementary material for: Influence of nutrient supply on plankton microbiome biodiversity and distribution in a coastal upwelling region
Source: Nat Commun. 2022 May 4;13:2448. doi: 10.1038/s41467-022-30139-4 (PMC9068609; doi:10.1038/s41467-022-30139-4)
Supplement: Supplementary file 6 — Reporting Summary [file 41467_2022_30139_MOESM6_ESM.pdf]

nature portfolio

Corresponding author(s): Chase James, Andrew Allen

Last updated by author(s): Mar 15, 2022

Reporting Summary

Nature Portfolio wishes to improve the reproducibility of the work that we publish. This form provides structure for consistency and transparency in reporting. For further information on Nature Portfolio policies, see our Editorial Policies and the Editorial Policy Checklist.

Statistics

For all statistical analyses, confirm that the following items are present in the figure legend, table legend, main text, or Methods section.

n/a	Confirmed	
x		The exact sample size (*n*) for each experimental group/condition, given as a discrete number and unit of measurement
	x	A statement on whether measurements were taken from distinct samples or whether the same sample was measured repeatedly
	x	The statistical test(s) used AND whether they are one- or two-sided<br>*Only common tests should be described solely by name; describe more complex techniques in the Methods section.*
	x	A description of all covariates tested
	x	A description of any assumptions or corrections, such as tests of normality and adjustment for multiple comparisons
	x	A full description of the statistical parameters including central tendency (e.g. means) or other basic estimates (e.g. regression coefficient) AND variation (e.g. standard deviation) or associated estimates of uncertainty (e.g. confidence intervals)
	x	For null hypothesis testing, the test statistic (e.g. *F*, *t*, *r*) with confidence intervals, effect sizes, degrees of freedom and *P* value noted<br>*Give P values as exact values whenever suitable.*
x		For Bayesian analysis, information on the choice of priors and Markov chain Monte Carlo settings
x		For hierarchical and complex designs, identification of the appropriate level for tests and full reporting of outcomes
x		Estimates of effect sizes (e.g. Cohen's *d*, Pearson's *r*), indicating how they were calculated

Our web collection on statistics for biologists contains articles on many of the points above.

Software and code

Policy information about availability of computer code

Data collection	No software was used for data collection
Data analysis	The code for this study is located at https://github.com/ChaseCJames/NCOG_Spatial_Environ.<br>DOI: 10.5281/zenodo.6359865<br>Analysis was done in R version 4.1.0<br>R packages used in this analysis include: SOMbrero v1.3-1, tidyverse v1.3.1, lubridate v1.710, vegan v2.5-7, spatialEco v 1.3-7, geosphere 1.5-10, viridis v0.6.1, ggmap v3.0.0, ggsn v0.5.0, patchwork v1.1.1, cmocean v0.3.-1, ncdf4 v1.17, scales v1.1.1, mgcv v1.8-35, stats v4.1.0

For manuscripts utilizing custom algorithms or software that are central to the research but not yet described in published literature, software must be made available to editors and reviewers. We strongly encourage code deposition in a community repository (e.g. GitHub). See the Nature Portfolio guidelines for submitting code & software for further information.

Data

Policy information about availability of data

All manuscripts must include a data availability statement. This statement should provide the following information, where applicable:

- Accession codes, unique identifiers, or web links for publicly available datasets
- A description of any restrictions on data availability
- For clinical datasets or third party data, please ensure that the statement adheres to our policy

The 16S rDNA raw reads have been deposited at NCBI under Bioproject IDs PRJNA555783 [https://www.ncbi.nlm.nih.gov/bioproject/PRJNA555783], PRJNA665326 [https://www.ncbi.nlm.nih.gov/bioproject/?term=PRJNA665326] and PRJNA804265 [https://www.ncbi.nlm.nih.gov/bioproject/?term=PRJNA804265] and Biosample accession nos. SAMN25705811-SAMN25706151, SAMN16250568-SAMN16251083, and SAMN25756929-SAMN25757078 and for the 2014-2016, 2017-2019, and

2020 periods respectively. The 18S rDNA raw reads have been deposited at NCBI under Bioproject IDs PRJNA555783 [https://www.ncbi.nlm.nih.gov/bioproject/PRJNA555783], PRJNA665326 [https://www.ncbi.nlm.nih.gov/bioproject/?term=PRJNA665326], and PRJNA804265 [https://www.ncbi.nlm.nih.gov/bioproject/?term=PRJNA804265] and Biosample accession nos. SAMN25710021-SAMN25710361, SAMN16251281-SAMN16251796, and SAMN25757352-SAMN25757501 for the 2014-2016, 2017-2019, and 2020 periods respectively.
Tara Oceans and Tara Polar 18Sv9 sequences can be found at the European Nucleotide Archive under the project accession IDs PRJEB6610 [https://www.ebi.ac.uk/ena/browser/view/PRJEB6610] and PRJEB9737 [https://www.ebi.ac.uk/ena/browser/view/PRJEB9737] respectively.
Associated sample metadata are provided in the Supplementary Data 2 file.

Field-specific reporting

Please select the one below that is the best fit for your research. If you are not sure, read the appropriate sections before making your selection.

☐ Life sciences ☐ Behavioural & social sciences ☒ Ecological, evolutionary & environmental sciences

For a reference copy of the document with all sections, see nature.com/documents/nr-reporting-summary-flat.pdf

Ecological, evolutionary & environmental sciences study design

All studies must disclose on these points even when the disclosure is negative.

Study description

Molecular and environmental data were collected quarterly on CalCOFI cruises (winter, spring, summer, and fall) within the Southern California Current region. At each station, seawater was collected near the surface (10m) and at the depth of the deep chlorophyll maximum, which varies in time and space

Research sample

For molecular data, approximately 0.5 - 2L of seawater was filtered through a 0.22 µm Sterivex-GP filter unit (MilliporeSigma, Burlington, MA, USA) for all DNA samples. Samples were immediately sealed with a sterile luer-lock plug and hematocrit sealant, wrapped in aluminum foil, and flash frozen in liquid nitrogen. DNA was extracted with the NucleoMag Plant Kit for DNA purification (Macherey-Nagel, Düren, Germany) on an epMotion 5057TMX (Eppendorf, Hamburg, Germany) as described here: https://dx.doi.org/10.17504/protocols.io.bc2hiyb6. DNA was assessed on a 1.8% agarose gel after extraction. The V4-V5 region of the 16S rRNA gene was targeted to identify prokaryotes while the V9 region of the 18S rRNA gene was targeted to identify eukaryotes within our samples. Combined these represent the populations of interest in this study. Full methods for environmental data collection and analysis can be found at: https://calcofi.org/references/methods

Sampling strategy

Two types of stations were sampled during this study: cardinal stations and productivity stations. Cardinal stations were sampled every cruise and occur on lines 80 (stations 55.0, 70.0, 80.0, 100.0), 81.8 (station 46.9) and 90 (stations 37.0, 53.0, 70.0, 90.0, 120.0) (Fig. 1a). Productivity stations, which measure 14C primary production at approximately local noon were also sampled. The locations of productivity stations vary from cruise to cruise depending on where the ship is located each day at approximately local noon. Productivity stations can overlap with cardinal stations during a given cruise if the ship is located at a cardinal station at local noon.

As described in the study description, two samples were taken per station. One sample was collected at the surface (10m) while another was collected at the depth of the deep chlorophyll maximum. The number of samples in this study is the product of a continued time series (2014-2020) with more samples being collected each cruise. The 995 samples analyzed in this study provide a comprehensive spatial and temporal view of the Southern California Current region across seven-years.

Data collection

Data was collected by graduate students in the Allen Lab from 2014 to present. Anne Schulberg conducted most of the NCOG sampling from 2014-2020. Data collection occurs on quarterly CalCOFI cruises and individual samples are collected as described in the research sample description above. All samples were collected, processed, and recorded during each cruise. Metadata for samples is written on both the samples and logbook before being transfered to a datasheet.

Timing and spatial scale

Data within this manuscript spans from 2014-2020. Data were collected from San Diego to north of Point Conception and from nearshore to 500km offshore. The sampling grid used in this manuscript is known as the 75 Station Pattern (more details can be found here: https://calcofi.org/graphics/458-station-maps.html). Sampling occurs quarterly (winter, spring, summer, fall).

Data exclusions

Limited samples were taken beyond the 75 Station grid. As these samples do not span the entire time series (2014-2020) and were not sampled every season (winter, spring, summer, fall), we have excluded these samples from the study. Therefore, this study only focuses on samples within the 75 Station Pattern (https://calcofi.org/graphics/458-station-maps.html)

Reproducibility

Sampling procedure and protocol was consistent throughout the study. Protocols describing amplicon sequencing and analysis are available at: https://protocols.io/view/amplicon-library-preparation-bmuck6sw. As this study represents a long term ecological time series, individual samples are not replicable as they are sampled at discrete time points and locations.

Randomization

Samples were collected following the grid of CalCOFI and are therefore not randomized. Sampling procedure was consistent throughout the study.

Blinding

Blinding was not relevant to our study. This study is part of a long term spatio-temporal monitoring program, CalCOFI, which has operated for over 70 years

Did the study involve field work? ☒ Yes ☐ No

Field work, collection and transport

Field conditions	Collection of samples occurred during quarterly CalCOFI cruises. The 75 Station Pattern represents large spatial gradients in temperature, upwelling, and productivity from the nutrient rich nearshore to the oligotropgic offshore. Changes in ocean stratification, upwelling, and temperature varied both seasonally and interannually from 2014-2020. These spatial and temporal gradients structure the area and the composition of the ecological communities that exist within the Southern California Current region.
Location	Sampling occured in the 75 Station Pattern of the CalCOFI grid (https://calcofi.org/graphics/458-station-maps.html). Stations span latitudinally from 29.75°N-35°N and longitudinally from 124°W-117°W
Access & import/export	Sampling was done on Scripps Institution of Oceanography or NOAA vessels quarterly from 2014-2020. Cruises are in compliance with local, national, and international laws.
Disturbance	Disturbances to the environment are minimal and are no greater than the disturbance caused by any sea-going vessel. All sampling gear was retrieved from the environment after sampling.

Reporting for specific materials, systems and methods

We require information from authors about some types of materials, experimental systems and methods used in many studies. Here, indicate whether each material, system or method listed is relevant to your study. If you are not sure if a list item applies to your research, read the appropriate section before selecting a response.

Materials & experimental systems

n/a	Involved in the study	
☒	☐	Antibodies
☒	☐	Eukaryotic cell lines
☒	☐	Palaeontology and archaeology
☐	☒	Animals and other organisms
☒	☐	Human research participants
☒	☐	Clinical data
☒	☐	Dual use research of concern

Methods

n/a	Involved in the study	
☒	☐	ChIP-seq
☒	☐	Flow cytometry
☒	☐	MRI-based neuroimaging

Animals and other organisms

Policy information about studies involving animals; ARRIVE guidelines recommended for reporting animal research

Laboratory animals	No laboratory animals were used in this study
Wild animals	This sampling only explored eukaryotic and prokaryotic protists filtered from seawater. Samples were filtered then immediately placed in liquid nitrogen.
Field-collected samples	Samples were collected on board cruises, filtered, and then immediately placed in liquid nitrogen.
Ethics oversight	No ethical guidance or approval was necessary for this study.

Note that full information on the approval of the study protocol must also be provided in the manuscript.

March 2021
